# Supplementary material for: TRIAD1 and HHARI bind to and are activated by distinct neddylated Cullin-RING ligase complexes
Source: EMBO J. 2013 Sep 27;32(21):2848–60. doi: 10.1038/emboj.2013.209 (PMC3817463; doi:10.1038/emboj.2013.209)
Supplement: Supplementary Data [file emboj2013209s1.pdf]

Supplementary Material

TRIAD1 and HHARI bind to and are activated by distinct neddylated Cullin RING ligase complexes

Ian R. Kelsall[1,2], David M. Duda[3], Jennifer L. Olszewski[3], Kay Hofmann[4], Axel Knebel[1,2], Frédéric Langevin[5], Nicola Wood[1,2], Melanie Wightman[1,2], Brenda A. Schulman[3], Arno F. Alpi[1,2,*]

[1]Scottish Institute for Cell Signalling, College of Life Sciences, University of Dundee, DD1 5EH, UK. [2]MRC Protein Phosphorylation and Ubiquitylation Unit, College of Life Sciences, University of Dundee, DD1 5EH, UK. [3]Department of Structural Biology & Howard Hughes Medical Institute, St. Jude Children's Research Hospital, Memphis, TN 38105, USA. [4]Institute of Genetics, University of Cologne, D-50674, Germany. [5]MRC Laboratory of Molecular Biology, Cambridge CB2 2QH, UK.

Running title: TRIAD1 and HHARI activation by Cullin RING ligases

*Corresponding author: a.f.alpi@dundee.ac.uk

Contents:

Supplementary Materials and Methods

5 Supplementary Figures

Supplementary Materials and Methods

Antibodies

The following primary antibodies were used for western blotting: anti-GFP (Roche), anti-ubiquitin FK2 (Enzo), anti-NEDD8 (Epitomics), anti-CUL4B and anti-FLAG-M2 (Sigma), anti-UBCH7 (Boston Biochem), anti-CUL1 and anti-CUL2 (Invitrogen), anti-RBX (Thermo Scientific), anti-Elongin-B and anti-Elongin-C (BioLegend), anti-p27^{Kip1}, anti-HA, and anti-β-actin (Cell Signaling Technology). Polyclonal sheep anti-TRIAD1 antibodies were generated by immunizing sheep with full-length TRIAD1 protein. Antibodies specific for Cullin 3 were raised in sheep against GST-Cullin 3 (554–768). Antibodies that recognize Cullin 4A were generated by immunizing sheep with GST-Cullin 4A (573–672). Antisera specific for Cullin 5 were raised in sheep immunized against GST-Cullin 5 (577–689).

Gene disruption of the chicken ARIH2 locus and generation of complementation cell lines

A gene-targeting construct was designed to remove exons 4 and 5 of the chicken *ARIH2* locus, thereby disrupting the N-terminal RING1 domain of the protein **(Supplementary Figure S5A)**. The 3′ arm of the targeting construct was amplified from chicken genomic DNA using the PCR primers AAGAATTCCAGGAACTTGTTGGCTGGTGCT-TGACTCTT and AAGGTACCAGAGTTCACCAGCCTTCCTGAATGCTAGTC and cloned into the *Eco*RI-*Kpn*I sites of the pBluescript II plasmid vector. The 5′ arm was TOPO cloned first into pCR-BluntII-TOPO (Invitrogen) using the PCR primers

GGGCCAGGAGGGAATTTCTTTGTGATAGGA and CTCGCATCACTTCACTT-CTGCCTATGCTAC and then subcloned into the *Not*I-*Bam*HI sites of the 3′ arm-containing pBluescript II plasmid. A drug selection cassette, conferring resistance to either neomycin or puromycin was then inserted into this same *Bam*HI site to complete the targeting construct. Chicken DT40 cells were sequentially transfected by electroporation with Gg*ARIH2-puro* (first allele) and Gg*ARIH2-neo* (second allele) targeting constructs and selected in the presence of the appropriate selection agent. Targeted integrations were detected by Southern blot analysis of *BamHI/NdeI*-digested genomic DNA **(Supplementary Figure S5B)** and PCR screening.

To generate DT40 cell lines expressing human TRIAD1 (WT) and human TRIAD1 (C300A), the relevant constructs were subcloned first into the *Sma*I site of pExpress then into the *Spe*I site of pLoxBsr (Arakawa et al, 2001). These TRIAD1 transgenes were stably integrated into Gg*ARIH2*$^{-/-}$ DT40 cells as described previously.

Supplementary references

Arakawa H, Lodygin D, Buerstedde JM (2001) Mutant loxP vectors for selectable marker recycle and conditional knock-outs. *BMC biotechnology* 1: 7

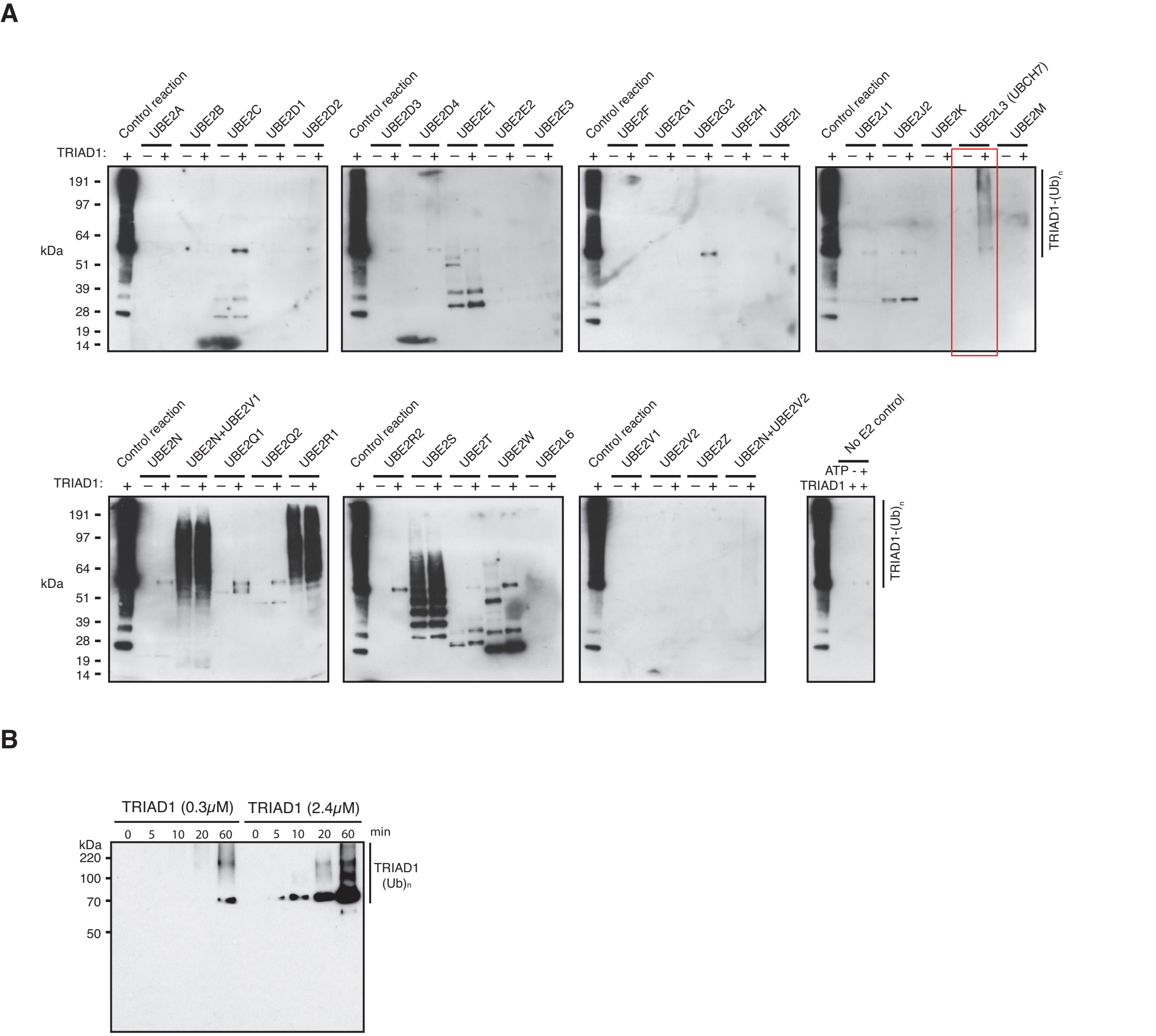

Supplementary Figure S1 Identification of E2 conjugating enzymes that facilitate TRIAD1 auto-ubiquitylation. **(A)** An *in vitro* screen of 34 different E2 enzymes was carried out using Ubiquigent's E2^{scan} kit together with 1 μM TRIAD1. Reaction products were analyzed by immunoblotting with anti-ubiquitin antibody. Only UBCH7 showed appreciable TRIAD1-dependent activity (shown boxed in red). To allow comparison of data across multiple western blots a control reaction was performed and loaded on all gels, as indicated. This control reaction contained TRIAD1 and our own UBCH7 and UBE1 protein preparations under reaction conditions otherwise identical to those of the E2^{scan} kit. **(B)** *in vitro* auto-ubiquitylation reaction with 0.3 or 2.4 μM TRIAD1 and 0.6 μM UBCH7, measured at the indicated timepoints by immunoblotting with anti-ubiquitin antibody.

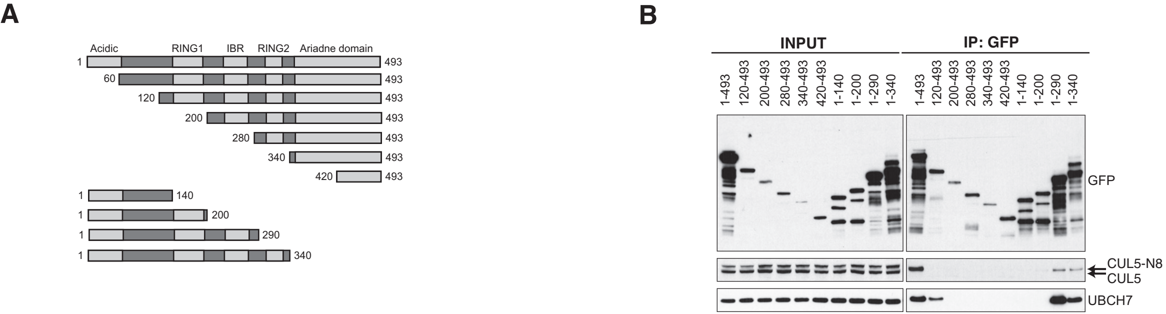

Supplementary Figure S2 The acidic N terminus of TRIAD1 is essential for its interaction with CUL5 **(A)** Schematic representation of TRIAD1 truncations analysed for CUL5 binding. **(B)** Anti-GFP immunoprecipitates (IP: GFP) and cell lysates (INPUT) from HEK293 cells stably expressing the indicated GFP-tagged TRIAD1 constructs were immunoblotted with the indicated antibodies. CUL5-N8, neddylated CUL5.

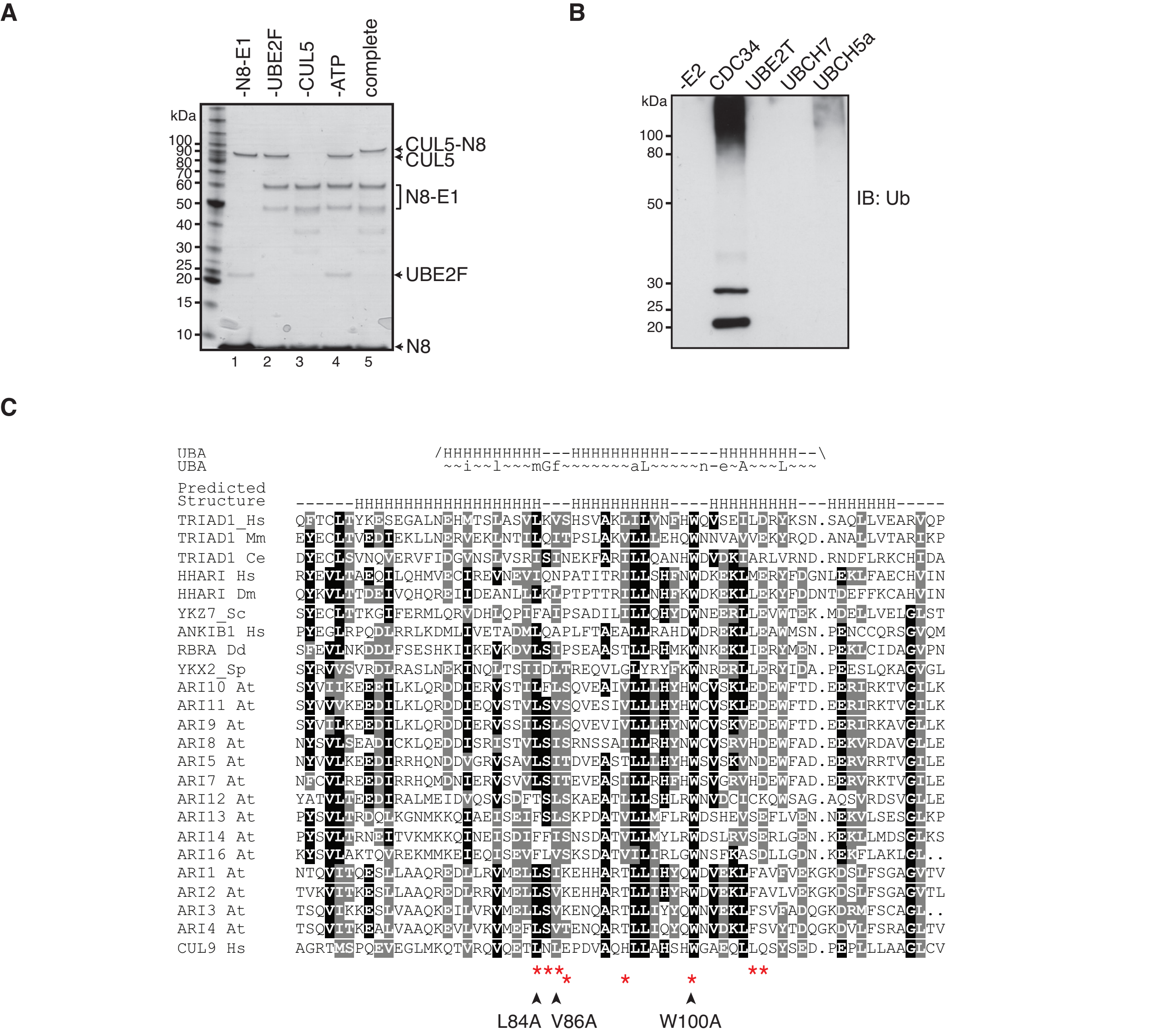

Supplementary Figure S3 TRIAD1/CUL5 interaction depends on NEDD8 **(A)** Complete neddylation reaction of CUL5–RBX2 complex (lane 5) and drop-out reactions (lane 1-4) were resolved by SDS-PAGE and stained with Coomassie blue. CUL5-N8, neddylated CUL5; N8, NEDD8. **(B)** Ubiquitylation reactions with neddylated CUL5–RBX2 complex in either the absence (–E2) or in the presence of the indicated E2 conjugating enzymes. Reaction products were resolved on SDS-PAGE and subjected to ubiquitin immunoblot analysis. **(C)** Alignment of predicted UBA-like domains in TRIAD1 and related Ariadne family members. The asterisks indicate notable conservation of residues in equivalent positions to those involved in ubiquitin binding in a conventional UBA domain (the upper row of asterisks) as well as conserved residues that do not lie within the canonical binding surface in a conventional UBA domain (the lower row of asterisks). The mutations introduced into TRIAD1 are indicated.

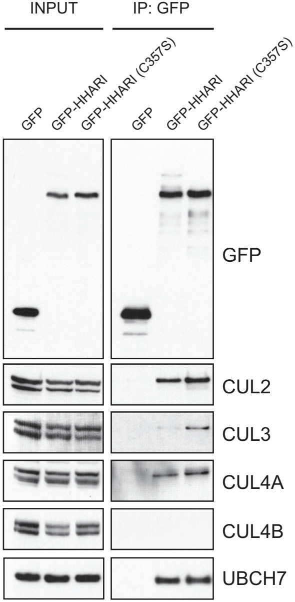

Supplementary Figure S4 HHARI associates with neddylated CRL complexes. GFP, GFP-tagged wild-type HHARI, or the RING2 mutant of HHARI containing the amino acid substitution C357S, were stably expressed in HEK293 cells and immunoprecipitated using anti-GFP agarose. The inputs and immunopellets (IP: GFP) were immunoblotted using the indicated antibodies.

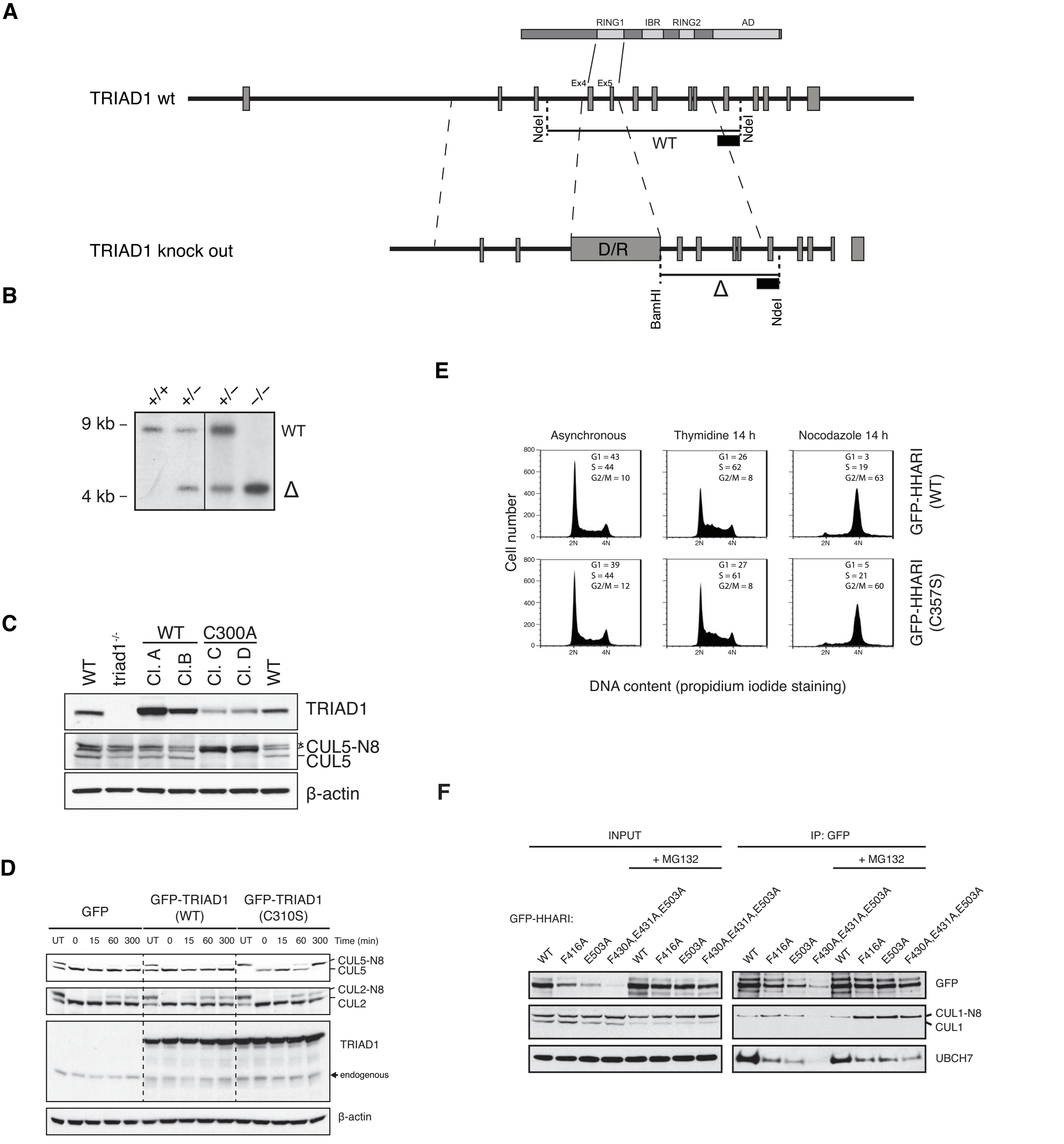

Supplementary Figure S5 TRIAD1 and HHARI impact on cullin neddylation in vivo. **(A)** Schematic presentation of the genomic locus of TRIAD1 with the exon configuration in correlation with TRIAD1 domain structures and the gene disruption construct removing exons 4–5. **(B)** Southern blot analysis of BamHI-NdeI digested genomic DNA from wild type (+/+), heterozygous (–/+) and homozygous (–/–) genotypes. wt, wild type locus; Δ, gene knock out locus; **(C)** Immunoblot analysis of TRIAD1 knock out DT40 cells (triad1-/-) complemented with human wild type TRIAD1 (WT, two independently derived clones Cl. A and Cl. B) or TRIAD1 with mutated RING2 domain (C300A, two independently derived clones Cl. C and Cl. D). β-actin serves as protein loading control. *indicates a non-specific band; **(D)** Cell lines as described in figure 7C, were treated for 3 hrs with 3 μM MLN4924 to completely abolish cullin neddylation. Cells were then washed to remove the inhibitor, allowed to recover in fresh medium for the indicated time periods and then whole cell lysates were subjected to immunoblot analyses with the indicated antibodies. **(E)** Flow cytometry cell cycle profiles of the indicated cell lines following treatment with thymidine or nocodazole. **(F)** The stability of the indicated GFP-HHARI constructs was analysed +/– MG132 by immunoblotting following anti-GFP immunoprecipitation. CUL2-N8, neddylated CUL2; CUL5-N8, neddylated CUL5.
